# Changes in Performance Measures and Service Volume at US Federally Qualified Health Centers During the COVID-19 Pandemic

**DOI:** 10.1001/jamahealthforum.2023.0351

**Published:** 2023-04-07

**Authors:** Megan B. Cole, Eun Kyung Lee, Bianca K. Frogner, Brad Wright

**Affiliations:** 1 Department of Health Law, Policy & Management, Boston University School of Public Health, Boston, Massachusetts; 2 Department of Family Medicine, University of Washington School of Medicine, Seattle; 3 Department of Health Services Policy and Management, Arnold School of Public Health, University of South Carolina, Columbia

## Abstract

**Importance:**

Stay-at-home orders, site closures, staffing shortages, and competing COVID-19 testing and treatment needs all potentially decreased primary care access and quality during the COVID-19 pandemic. These challenges may have especially affected federally qualified health centers (FQHCs), which serve patients with low income nationwide.

**Objective:**

To examine changes in FQHCs’ quality-of-care measures and visit volumes in 2020 to 2021 vs prepandemic.

**Design, Setting, and Participants:**

This cohort study used a census of US FQHCs to calculate changes in outcomes between 2016 and 2021 using generalized estimating equations.

**Main Outcomes and Measures:**

Twelve quality-of-care measures and 41 visit types based on diagnoses and services rendered, measured at the FQHC-year level.

**Results:**

A total of 1037 FQHCs were included, representing 26.6 million patients (63% 18-64 years old; 56% female) in 2021. Despite upward trajectories for most measures prepandemic, the percentage of patients served by FQHCs receiving recommended care or achieving recommended clinical thresholds showed a statistically significant decrease between 2019 and 2020 for 10 of 12 quality measures. For example, declines were observed for cervical cancer screening (−3.8 percentage points [pp]; 95% CI, −4.3 to −3.2 pp), depression screening (−7.0 pp; 95% CI, −8.0 to −5.9 pp), and blood pressure control in patients with hypertension (−6.5 pp; 95% CI, −7.0 to −6.0 pp). By 2021, only 1 of these 10 measures returned to 2019 levels. From 2019 to 2020, 28 of 41 visit types showed a statistically significant decrease, including immunizations (incidence rate ratio [IRR], 0.76; 95% CI, 0.73-0.78), oral examinations (IRR, 0.61; 95% CI, 0.59-0.63), and supervision of infant or child health (IRR, 0.87; 95% CI, 0.85-0.89); 11 of these 28 visits approximated or exceeded prepandemic rates by 2021, while 17 remained below prepandemic rates. Five visit types increased in 2020, including substance use disorder (IRR, 1.07; 95% CI, 1.02-1.11), depression (IRR, 1.06; 95% CI, 1.03-1.09), and anxiety (IRR, 1.16; 95% CI, 1.14-1.19); all 5 continued to increase in 2021.

**Conclusions and Relevance:**

In this cohort study of US FQHCs, nearly all quality measures declined during the first year of the COVID-19 pandemic, with most declines persisting through 2021. Similarly, most visit types declined in 2020; 60% of these remained below prepandemic levels in 2021. By contrast, mental health and substance use visits increased in both years. The pandemic led to forgone care and likely exacerbated behavioral health needs. As such, FQHCs need sustained federal funding to expand service capacity, staffing, and patient outreach. Quality reporting and value-based care models must also adapt to the pandemic’s influence on quality measures.

## Key Points

**Question:**

How did quality measures and visit volumes change at federally qualified health centers (FQHCs) in 2020 to 2021 compared with before the COVID-19 pandemic?

**Findings:**

In this cohort study of 1037 US FQHCs, 10 of 12 quality measures declined in 2020—losses that largely persisted into 2021. Most FQHC visit types declined in 2020, although mental health and substance use visits increased; by 2021, some visit volumes remained below prepandemic levels, while others returned to or exceeded prepandemic levels.

**Meaning:**

Sustained federal funding to FQHCs is needed to compensate for missed care during the COVID-19 pandemic, and quality-dependent value-based care models must adapt to the pandemic’s influence on quality measures.

## Teaser

This cohort study examines changes in federally qualified health centers’ quality-of-care measures and visit volumes in 2020 to 2021 compared with before the COVID-19 pandemic.

## Introduction

COVID-19 rapidly changed the primary care landscape, with stay-at-home orders, concerns about contracting SARS-CoV-2, site closures, staffing shortages, and competing COVID-19 testing and treatment needs all potentially decreasing access to and quality of primary care services.^1,2,3,4,5,6,7^ These challenges may have especially affected federally qualified health centers (FQHCs),^8,9^ which serve nearly 30 million patients with low income from marginalized communities across the US.^10^

Federally qualified health centers are community-based centers providing preventive, chronic disease management, mental health and substance use, and enabling services to patients without regard for ability to pay.^11^ Federally qualified health centers are seen as trusted and accessible entities that are well positioned to help mitigate health inequities by delivering high-quality, culturally competent care.^12,13,14^ They play a critical role in the US health system by providing access to care for populations who would otherwise lack it, and their patient population—which is majority non-White, low income, and Medicaid enrolled or uninsured—represents groups disproportionately and inequitably affected by COVID-19.^15,16^

Ensuring both continuity and quality of care during the COVID-19 pandemic is particularly important for patients in FQHCs, as to mitigate further disparities in health outcomes. To support FQHCs’ capacity to provide care, more than $9 billion in federal grant funding was made available exclusively to FQHCs during the COVID-19 pandemic,^17^ while FQHCs were also eligible for other sources of federal relief funding.^18^ Meanwhile, many FQHCs rapidly adopted telehealth capabilities to ensure continuity of care, with 95% of FQHCs using at least some telehealth during the pandemic’s first year.^19^ Despite these supports, FQHC use may have been affected by workforce challenges^20^ combined with changes in patient care-seeking behavior, such as avoiding in-person care to reduce exposure risk.

Collectively, these factors may have led to reduced use of important health services at FQHCs. Fewer visits, coupled with less available staff time, competing COVID-19 testing demands, limited access to specialists, and substitution of in-person visits for telehealth visits, may have also led to diminished quality-of-care measures, particularly for process measures that depend on receiving a recommended service. However, only 1 known study^21^ has examined changes in quality-of-care measures at US FQHCs following the onset of the COVID-19 pandemic. That study was limited to 6 measures in 2020, and the extent to which service volume changed across US FQHCs due to pandemic-associated disruptions is unknown. Understanding these changes is important as we move forward and develop policies and programs that compensate for potential care losses, including missed preventive services such as cancer screenings and inadequate treatment of chronic conditions. Understanding how quality and service use has changed during the COVID-19 pandemic also has implications for value-based payment models and other delivery models that depend on measured quality. To address these knowledge gaps, the objective of this study was to examine changes in quality-of-care measures and visit volumes at FQHCs during the first 2 years of the pandemic (2020-2021) vs before the pandemic, using a census of US FQHCs.

## Methods

### Data Sources and Study Population

Data were collected from the 2016 to 2021 Health Resources and Services Administration Uniform Data System (UDS), which captured data through December 31, 2021. Data from UDS are reported annually by every federally funded Section 330 FQHC in the US, as reported at the FQHC level, and include information on patient demographics (eg, age, sex, self-reported race and ethnicity), services rendered, quality-of-care measures, and organizational characteristics.^10^ Of the 1373 FQHCs operating in 2021, we excluded those without Section 330(e) Community Health Center funding (n = 68), located in US territories (n = 32), that were newly established between 2016 and 2020 (n = 218; see eTable 2 in Supplement 1 for characteristics of these excluded FQHCs), and with highly missing data (n = 18). The final sample included 1037 FQHCs per year, representing 26.6 million patients in 2021.

Boston University’s institutional review board deemed the study exempt from review and waived need for informed consent because data were publicly available. This study followed the Strengthening the Reporting of Observational Studies in Epidemiology (STROBE) reporting guidelines.

### Study Outcomes

We examined 2 primary sets of study outcomes: quality and visit volume measures. First, we examined 12 quality-of-care Healthcare Effectiveness Data and Information Set (HEDIS) measures, as further defined by the Health Resources and Services Administration in the UDS Manual (eTable 1 in Supplement 1).^22^ These included 9 process measures (cervical cancer screening, colorectal cancer screening, body mass index [BMI] screening and follow-up plan in adults, weight assessment and counseling for nutrition and physical activity in children, depression screening and follow-up plan, tobacco use screening and cessation intervention, early entry into prenatal care, use of aspirin or antiplatelet in ischemic vascular disease, and dental sealants for children) and 3 intermediate outcome measures (blood pressure control in patients with hypertension, blood glucose control in patients with diabetes, and normal birth weight for infants born to pregnant patients). All quality measures were reported as the percentage of eligible patients achieving the measure among patients with at least 1 medical visit during the measurement year. Collectively, these measures represented the universe of quality measures consistently reported in the UDS since 2016—4 years prior to the start of the COVID-19 pandemic. Second, we examined 41 visit types based on diagnoses (eg, asthma, diabetes, depression, alcohol use disorder) or services rendered (eg, HIV testing, immunizations, eye examination, oral examination), measured as the total number of visits per FQHC per year. This represents the universe of visit measures reported in each year from 2016 through 2021, excluding measures with fewer than 50 visits per FQHC per year on average. Detailed specifications on visit definitions are provided in the UDS Manual.^22^ Finally, as a secondary measure of FQHC volume, we examined the unique number of patients served.

### Statistical Analysis

Using FQHC-year as the unit of analysis, we compared year-over-year changes in outcomes between 2016 and 2021. To do so, we used generalized estimating equations with autoregressive correlation structures, state fixed effects, and FQHC-level clustering to account for repeated measures. We examined quality measures using a linear specification and adjusted for the percentage of FQHC patients who were 18 to 64 years old, were female, were from a racial or ethnic minoritized group, were uninsured, had income below the federal poverty level, spoke a primary language other than English, and were from a rural area. We examined visit volumes using a negative binomial specification with a log link to account for overdispersion in visit outcomes. To calculate year-over-year changes for each outcome, we estimated these models using 2016 to 2021 data, treating categorical year as the independent variable, with 2019 as the reference year. While we emphasize 2020 and 2021 coefficients (interpreted relative to 2019) to illustrate pandemic-era changes, we also report year-over-year changes from 2016 to 2018 (relative to 2019) to capture secular trends leading up to the 2020 and 2021 pandemic period.

All *P *values were 2-tailed, with a priori statistical significance of α = .05. Analyses were performed using Stata, version 17.0 (StataCorp).

## Results

### Study Population

The final study sample included 1037 unique FQHCs per year, serving 26.6 million patients in 2021. Of these patients in 2021, 63% were 18 to 64 years old; 56% identified as female; 19% were non-Hispanic Black and 28% were Hispanic (any race) based on self-reported race and ethnicity; 63% had incomes below the federal poverty level; 43% were enrolled in Medicaid; 22% were uninsured; and 44% were served by FQHCs located in rural areas (Table 1). The composition of the study population was similar in the years 2016 to 2019 vs 2020 to 2021, although a smaller proportion of patients at FQHCs were children in 2020 and 2021 compared with prior years.

**Table 1.** Characteristics of US Federally Qualified Health Centers (FQHCs) by Study Year (N = 1037)

Characteristic	Patients, %					
	2016 (representing 23.0 million)	2017 (representing 24.1 million)	2018 (representing 25.2 million)	2019 (representing 26.2 million)	2020 (representing 25.3 million)	2021 (representing 26.6 million)
Age group						
Children (<18 y)	27.2	27.1	27.1	27.1	23.1	24.1
Adults (18-64 y)	63.1	62.7	62.2	61.6	63.4	61.9
Elderly (≥65 y)	9.7	10.2	10.8	11.3	13.5	14.0
Sex						
Female	56.7	56.7	56.7	56.6	56.7	56.4
Male	43.3	43.3	43.3	43.4	43.3	43.6
Race and ethnicity						
Black, non-Hispanic	19.4	19.3	19.1	18.9	18.7	18.9
Hispanic, any race	26.5	26.7	27.1	27.4	27.8	28.1
White, non-Hispanic	43.2	42.9	42.4	42.0	42.0	41.1
Other race, non-Hispanic^a^	10.9	11.1	11.3	11.7	11.5	11.8
Primary language other than English	17.0	16.9	16.9	17.7	18.8	19.1
Insurance coverage type						
Medicaid	43.1	43.1	42.7	42.0	41.1	42.9
Uninsured	25.5	24.9	24.6	24.6	23.6	22.1
Private	19.9	20.2	20.7	21.2	22.5	22.2
Medicare	10.7	11.0	11.4	11.6	12.2	12.3
Other	0.8	0.7	0.7	0.6	0.7	0.6
Poverty level						
<100% Of federal poverty level	66.3	65.6	64.5	64.1	64.2	63.2
100%-200% Of federal poverty level	24.0	24.2	25.0	25.1	25.1	25.0
>200% Of federal poverty level	9.6	10.2	10.5	10.8	10.7	11.8
Rural	44.7	44.7	43.3	40.8	44.3	44.3
Without housing	7.6	7.7	7.5	7.3	8.0	7.4
Unique patients served/FQHC, mean (SD)	22 189 (24 719)	23 259 (25 786)	24 287 (27 318)	25 307 (28 897)	24 424 (29 006)	25 667 (29 743)

^a^ Other race includes patients who identify as American Indian or Alaska Native, Asian, Native Hawaiian or Other Pacific Islander, or more than 1 race. These categories were grouped together owing to small sample sizes.

### Changes in Quality-of-Care Measures

**Figure 1.**
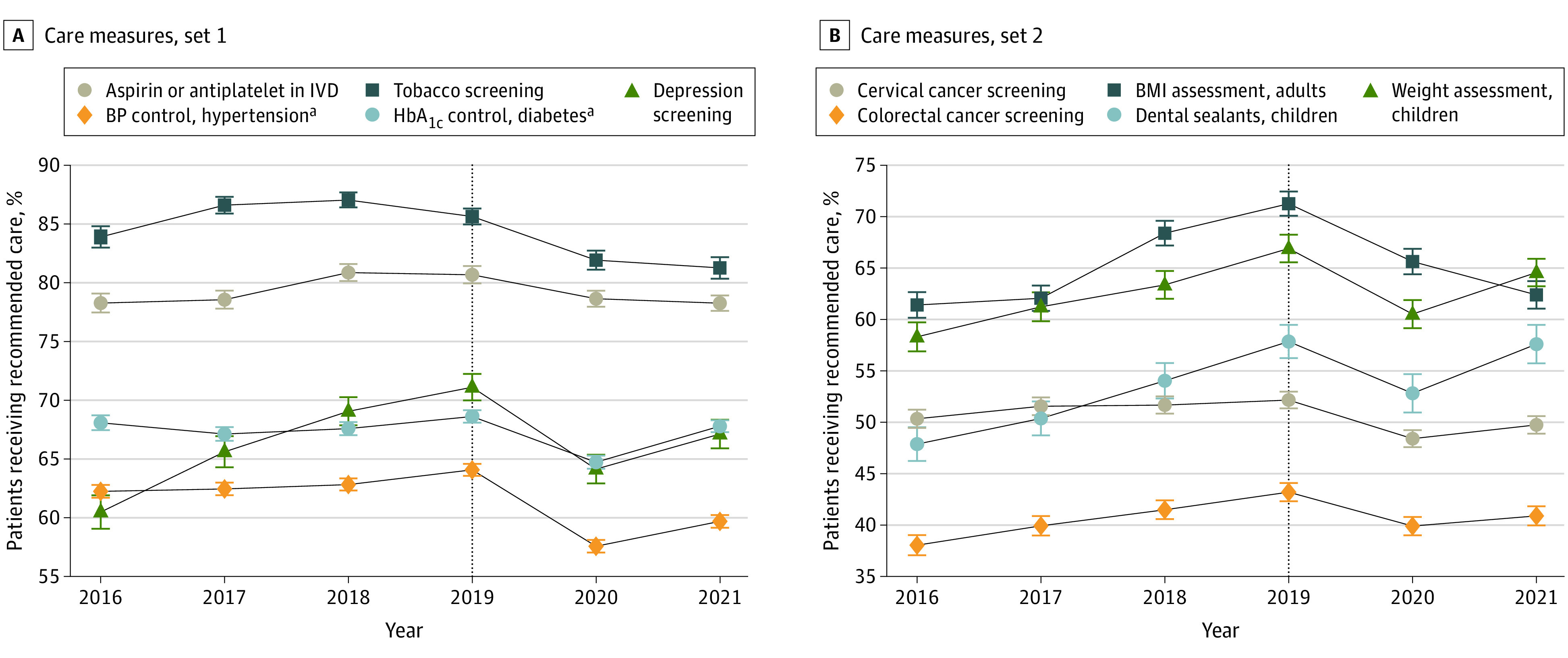
Trends in Quality-of-Care Measures at US Federally Qualified Health Centers, 2016-2021 BMI indicates body mass index; HbA_1c_, hemoglobin A_1c_; IVD, ischemic vascular disease. ^a^Blood pressure (BP) and HbA_1c_ control measures require (1) clinical control (ie, most recent BP <140/90 mm Hg and HbA_1c_ <9%) and (2) having a documented measurement in the study period. Patients who qualify for the denominator but who do not have a documented measurement are classified as not having control.

Despite upward trajectories for most measures from 2016 to 2019, the percentage of patients served by FQHCs receiving recommended care or meeting the recommended clinical threshold showed a statistically significant decrease for 10 of 12 quality-of-care measures from 2019 to 2020 (Figure 1A and B). For instance, in 2020, declines were observed for cervical cancer screenings (−3.8 percentage points [pp]; 95% CI, −4.3 to −3.2 pp), BMI assessment in adults (−5.6 pp; 95% CI, −6.5 to −4.7 pp) and in children (−6.4 pp; 95% CI, −7.5 to −5.3 pp), colorectal cancer screenings (−3.3 pp; 95% CI, −4.0 to −2.6 pp), depression screening (−7.0 pp; 95% CI, −8.0 to −5.9 pp), dental sealants for children (−5.0 pp; 95% CI, −6.7 to −3.4 pp), blood pressure control in patients with hypertension (−6.5 pp; 95% CI, −7.0 to −6.0 pp), and blood glucose control in patients with diabetes (−3.9 pp; 95% CI, −4.4 to −3.3 pp) (eTable 3 in Supplement 1).

By 2021, only 1 of the 10 quality measures that declined in 2020—dental sealants for children—had returned to 2019 levels. Of the remaining 9 quality measures, 6 measures started to directionally improve in 2021 compared with 2020 (eg, depression screening, blood pressure control), while 3 measures continued to directionally decline (eg, tobacco screening, BMI assessment in adults). In contrast, the percentage of pregnant patients delivering infants with normal birth weight increased slightly in 2020 (1.2 pp; 95% CI, 0.4-2.0 pp), with no statistically significant change in 2021 compared with 2019. There was not a statistically significant change in the percentage of pregnant patients with early entry to prenatal care in 2020 or 2021 (eTable 3 and eFigures 1 and 2 in Supplement 1).

### Changes in Visit Volumes

**Figure 2.**
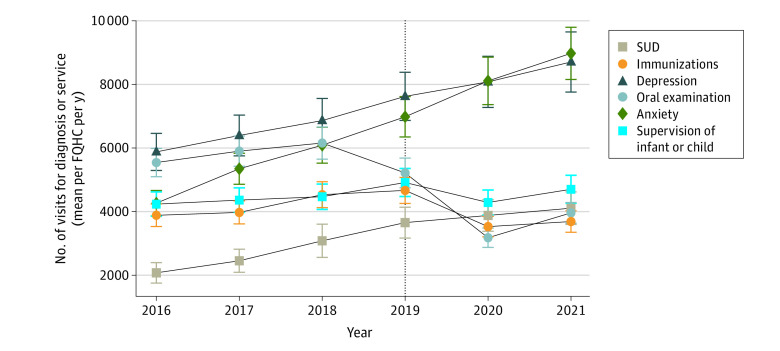
Trends in Select Visit Volumes at US Federally Qualified Health Centers (FQHCs), 2016-2021 Error bars represent 95% CIs, and the dashed line represents the reference year. SUD indicates substance use disorder.

Despite upward trajectories for most visit types from 2016 to 2019, 28 of 41 visit types decreased at FQHCs from 2019 to 2020 (Table 2). For instance, visits for immunizations (incidence rate ratio [IRR], 0.76; 95% CI, 0.73-0.78), oral examinations (IRR, 0.61; 95% CI, 0.59-0.63), and supervision of infant or child health (IRR, 0.87; 95% CI, 0.85-0.89) all showed a statistically significantly decrease (Figure 2). By 2021, 17 of the 28 visit types that declined in 2020 remained below 2019 levels; 6 of 28 continued to directionally decline in 2021 (eg, visits for hepatitis C), and 11 of 28 directionally improved in 2021 but remained lower than 2019 rates (eg, visits for child lead testing). Only 11 of the 28 visit types that declined in 2020 approximated or exceeded prepandemic levels in 2021; 8 of 28 were statistically similar to 2019 rates (eg, visits for pap testing), and 3 of 28 exceeded 2019 rates (eg, visits for diabetes) (Table 2).

**Table 2.** Changes in US Federally Qualified Health Center (FQHC) Visit Volumes by Diagnosis and Service Type, 2016-2021

Variable	IRR (95% CI)^a^				
	Prior to COVID-19 pandemic			During COVID-19 pandemic	
	2016 (vs 2019)	2017 (vs 2019)	2018 (vs 2019)	2020 (vs 2019)	2021 (vs 2019)
Visits with diagnosis					
HIV	0.95 (0.87-1.03)	0.91 (0.84-0.98)^b^	1.00 (0.92-1.08)	0.96 (0.93-1.00)^b^	0.91 (0.86-0.96)^b^
Sexually transmitted infection	0.83 (0.80-0.87)^b^	1.01 (0.94-1.08)	0.99 (0.96-1.02)	1.12 (1.08-1.16)^b^	1.27 (1.21-1.33)^b^
Hepatitis B	0.89 (0.80-0.98)^b^	0.91 (0.85-0.98)^b^	1.02 (0.97-1.07)	0.89 (0.83-0.97)^b^	0.81 (0.75-0.89)^b^
Hepatitis C	1.05 (0.98-1.13)	1.03 (0.97-1.10)	1.00 (0.96-1.03)	0.83 (0.76-0.89)^b^	0.73 (0.70-0.76)^b^
Asthma	0.93 (0.91-0.96)^b^	0.97 (0.95-0.99)^b^	0.98 (0.97-0.99)^b^	0.64 (0.62-0.96)^b^	0.94 (0.91-0.97)^b^
Bronchitis	0.91 (0.88-0.94)^b^	0.95 (0.93-0.98)^b^	0.96 (0.94-0.99)^b^	0.90 (0.86-0.93)^b^	0.93 (0.89-0.98)^b^
Abnormal breast results	0.36 (0.34-0.39)^b^	0.63 (0.60-0.67)^b^	0.91 (0.86-0.95)^b^	0.94 (0.91-0.98)^b^	1.09 (1.03-1.15)^b^
Abnormal cervical cancer results	0.83 (0.79-0.88)^b^	0.96 (0.91-1.01)	0.99 (0.95-1.03)	0.88 (0.84-0.92)^b^	0.98 (0.93-1.03)
Diabetes	0.89 (0.88-0.91)^b^	0.94 (0.92-0.96)^b^	0.97 (0.96-0.99)^b^	0.96 (0.95-0.98)^b^	1.05 (1.03-1.07)^b^
Heart disease	0.88 (0.84-0.91)^b^	0.90 (0.87-0.93)^b^	0.96 (0.93-0.99)^b^	0.93 (0.92-0.95)^b^	1.00 (0.98-1.03)
Hypertension	0.87 (0.85-0.88)^b^	0.90 (0.88-0.92)^b^	0.95 (0.94-0.97)^b^	0.94 (0.92-0.96)^b^	1.02 (1.00-1.04)^b^
Contact dermatitis	0.99 (0.95-1.03)	0.99 (0.95-1.02)	1.00 (0.97-1.03)	0.87 (0.85-0.88)^b^	0.90 (0.86-0.94)^b^
Dehydration	1.05 (0.89-1.24)	0.95 (0.89-1.01)	0.99 (0.94-1.05)	0.77 (0.73-0.82)^b^	0.73 (0.65-0.83)^b^
Overweight/obese	0.57 (0.53-0.62)^b^	0.72 (0.68-0.76)^b^	0.87 (0.84-0.91)^b^	0.79 (0.76-0.84)^b^	0.96 (0.90-1.02)
Otitis media	1.13 (1.09-1.18)^b^	1.03 (0.99-1.06)	0.97 (0.94-1.00)^b^	0.53 (0.50-0.56)^b^	0.51 (0.47-0.56)^b^
Perinatal/neonatal medical conditions	0.90 (0.85-0.95)^b^	0.92 (0.88-0.97)^b^	0.99 (0.94-1.04)	0.96 (0.93-0.99)^b^	0.92 (0.86-0.97)^b^
Abnormal development in children	1.50 (1.33-1.69)^b^	1.20 (1.07-1.34)^b^	1.04 (0.95-1.13)	0.83 (0.76-0.90)^b^	0.98 (0.87-1.09)
Alcohol use disorder	0.71 (0.67-0.77)^b^	0.79 (0.74-0.83)^b^	0.88 (0.85-0.91)^b^	1.05 (1.00-1.11)^b^	1.13 (1.08-1.19)^b^
Other substance use disorders	0.50 (0.46-0.55)^b^	0.62 (0.58-0.66)^b^	0.82 (0.72-0.95)^b^	1.07 (1.02-1.11)^b^	1.13 (1.06-1.19)^b^
Tobacco use	0.89 (0.82-0.98)^b^	0.96 (0.89-1.04)	1.00 (0.94-1.06)	0.90 (0.86-0.94)^b^	0.92 (0.88-0.97)^b^
Depression	0.77 (0.74-0.80)^b^	0.84 (0.81-0.87)^b^	0.90 (0.88-0.92)^b^	1.06 (1.03-1.09)^b^	1.14 (1.10-1.19)^b^
Anxiety	0.61 (0.58-0.64)^b^	0.77 (0.74-0.79)^b^	0.87 (0.85-0.89)^b^	1.16 (1.14-1.19)^b^	1.29 (1.25-1.32)^b^
Attention deficit disorder	0.77 (0.72-0.83)^b^	0.85 (0.81-0.90)^b^	0.91 (0.88-0.94)^b^	0.99 (0.94-1.04)	1.07 (1.03-1.13)^b^
Other mental health disorder	0.72 (0.68-0.77)^b^	0.78 (0.75-0.82)^b^	0.90 (0.87-0.92)^b^	1.03 (0.99-1.07)	1.14 (1.09-1.19)^b^
Visits with service rendered					
HIV testing	0.54 (0.49-0.59)^b^	0.74 (0.68-0.79)^b^	0.90 (0.86-0.95)^b^	0.95 (0.88-1.02)	1.29 (1.21-1.38)^b^
Hepatitis B testing	0.48 (0.41-0.57)^b^	0.69 (0.59-0.80)^b^	0.89 (0.78-1.02)	0.78 (0.67-0.91)^b^	1.14 (0.99-1.31)
Hepatitis C testing	0.49 (0.43-0.55)^b^	0.76 (0.69-0.84)^b^	0.86 (0.80-0.93)^b^	0.95 (0.86-1.05)	1.51 (1.34-1.69)^b^
Mammogram	0.69 (0.63-0.77)^b^	0.90 (0.84-0.96)^b^	0.99 (0.94-1.05)	1.01 (0.91-1.11)	1.49 (1.36-1.63)^b^
Pap testing	1.14 (1.09-1.18)^b^	1.08 (1.03-1.12)^b^	1.02 (0.99-1.05)	0.78 (0.75-0.81)^b^	1.01 (0.97-1.05)
Immunizations	0.83 (0.80-0.87)^b^	0.85 (0.82-0.89)^b^	0.97 (0.94-1.00)^b^	0.76 (0.73-0.78)^b^	0.79 (0.76-0.82)^b^
Flu vaccination	0.79 (0.75-0.83)^b^	0.83 (0.80-0.87)^b^	0.93 (0.89-0.97)^b^	0.95 (0.91-1.01)	0.82 (0.78-0.86)^b^
Contraception management	0.90 (0.87-0.94)^b^	0.94 (0.91-0.97)^b^	0.97 (0.95-0.99)^b^	0.91 (0.89-0.94)^b^	0.95 (0.91-1.00)
Health supervision of infant or child	0.86 (0.83-0.89)^b^	0.89 (0.87-0.91)^b^	0.91 (0.89-0.93)^b^	0.87 (0.85-0.89)^b^	0.96 (0.93-0.99)^b^
Child lead testing	0.75 (0.68-0.82)^b^	0.90 (0.83-0.98)^b^	0.91 (0.86-0.98)^b^	0.80 (0.74-0.87)^b^	0.87 (0.79-0.96)^b^
SBIRT	0.42 (0.31-0.56)^b^	0.55 (0.42-0.73)^b^	0.62 (0.49-0.78)^b^	0.96 (0.81-1.13)	0.85 (0.66-1.09)
Tobacco cessation services	0.73 (0.61-0.88)^b^	0.89 (0.73-1.09)	0.93 (0.82-1.06)	1.03 (0.89-1.20)	1.20 (1.03-1.40)^b^
Eye examination	0.84 (0.71-0.99)^b^	0.88 (0.80-0.98)^b^	0.96 (0.90-1.03)	0.75 (0.66-0.86)^b^	0.96 (0.84-1.09)
Oral examination	1.06 (1.02-1.10)^b^	1.13 (1.10-1.17)^b^	1.18 (1.15-1.21)^b^	0.61 (0.59-0.63)^b^	0.76 (0.72-0.80)^b^
Sealants	0.92 (0.86-1.00)^b^	0.92 (0.86-0.99)^b^	0.95 (0.91-1.00)^b^	0.55 (0.51-0.58)^b^	0.73 (0.67-0.80)^b^
Fluoride	0.82 (0.79-0.86)^b^	0.88 (0.84-0.91)^b^	0.94 (0.92-0.96)^b^	0.62 (0.59-0.64)^b^	0.78 (0.74-0.82)^b^
Dental restorative services	0.87 (0.80-0.95)^b^	0.92 (0.87-0.97)^b^	0.96 (0.93-1.00)^b^	0.58 (0.56-0.60)^b^	0.77 (0.69-0.86)^b^
Total patient volume					
Unique patients served	0.89 (0.88-0.91)^b^	0.93 (0.92-0.94)^b^	0.96 (0.96-0.97)^b^	0.96 (0.95-0.97)^b^	1.01 (0.99-1.03)

Abbreviations: IRR, incidence rate ratio; SBIRT, screening, brief intervention and referral to treatment.

^a^ The IRR represents the relative rate of number of visits per FQHC in each individual year compared with 2019. An IRR lower than 1.0 indicates a relative decrease in visits in that year compared with 2019. An IRR higher than 1.0 indicates a relative increase in that year compared with 2019.

^b^ *P* < .05.

Meanwhile, 5 of 41 visit types showed a statistically significant increase between 2019 and 2020, including visits for substance use disorder (IRR, 1.07; 95% CI, 1.02-1.11), depression (IRR, 1.06; 95% CI, 1.03-1.09), anxiety (IRR, 1.16; 95% CI, 1.14-1.19), and sexually transmitted infections (STIs) (IRR, 1.12; 95% CI, 1.08-1.16) (Figure 2 and Table 2). In 2021, all 5 of these visit types continued to directionally increase.

Finally, when examining changes in the total number of unique patients served per FQHC—a secondary FQHC volume outcome—the number of patients decreased from 2019 to 2020 (IRR, 0.96; 95% CI, 0.95-0.97). By 2021, the number of patients was statistically similar to 2019, although rates had increased year over year prior to 2019 (Table 2).

## Discussion

Using a census of US FQHCs in continuous operation during the study period, we found that nearly all quality-of-care HEDIS measures declined during the first year of the COVID-19 pandemic and nearly all of them remained below prepandemic rates through 2021. Visit volumes for preventive care, chronic disease care, follow-up care, eye examinations, and oral examinations also showed a statistically significant decrease in 2020. By 2021, some returned to prepandemic levels, while most remained below prepandemic levels. In contrast, visits for mental health and substance use diagnoses increased in 2020 and 2021. This study represents, to our knowledge, the first national examination of how important health services changed for patients with low income served by FQHCs during the first 2 years of the pandemic, a time of heightened vulnerability.

Declines in measured quality-of-care process measures were consistent with quality declines reported in other literature,^4,9,23,24^ including a recent study that found that FQHC preventive care quality measures declined in 2020.^21^ The present findings show that many of these declines persisted through 2021. Declines were likely due, in part, to patients coming in for fewer office visits and therefore missing recommended services. Temporary FQHC closures in the early months of the pandemic, or closures among laboratories and specialty clinics to which patients at FQHCs are referred, may have contributed to this.^25^ Even among patients with continuity of care, fewer services may have been received during visits due to other FQHC capacity constraints, such as competing COVID-19–related demands on staff time or having fewer staff available. Declines in hypertension control and diabetes control could be due to inadequate chronic disease management or due to lack of documented blood pressure and glucose levels, as both clinical control and documented measurement are required to comply with these 2 respective measures. The depression-screening quality measure experienced the largest absolute decrease in 2020, which could be due to fewer patients having visits coupled with disruptions and systematic changes to workflows, including the implementation of virtual care where patient screenings may be more difficult to administer. Declines in depression-screening rates are particularly concerning given that the pandemic coincided with substantial increases in depressive symptoms across the US and globally.^26,27^ Collectively, these observed declines in measured quality erased years of progress in quality-measure improvement, at least temporarily. These losses could take many years to recover, while forgone care could lead to delayed diagnoses or poor management of health conditions. These declines in quality measures also translate to millions of missed recommended services at the population level.

While declines in preventive care, chronic disease management, vision care, and dental service utilization have been widely reported during the COVID-19 pandemic,^28,29,30,31,32^ large declines within a low-income, racially diverse patient population could exacerbate health disparities among a population already experiencing preexisting inequities in access to and use of these services.^33,34,35,36^ While the COVID-19 pandemic was disruptive for everyone, historically marginalized populations—including those served by FQHCs—were disproportionately harmed.^15,16,33^ These same groups are likely to recover from the physical, mental, social, and economic harms of COVID-19 much more slowly, given the systematic inequities they face rooted in long-standing racism and classism. Thus, ensuring continued access to FQHC services during and after the COVID-19 pandemic—inclusive of health services and other social support services—may be especially important to mitigating further inequities.^37,38^

Unlike other service types, visit volumes for select behavioral health diagnoses and STIs increased in both 2020 and 2021 (vs 2019) and continued to follow their trajectory of growth based on historical trends. These observed increases are despite system capacity constraints and concurrent changes in patient care-seeking behavior. These increases are likely due, in part, to increased demand for both mental health and substance use services, as behavioral health needs were exacerbated by the indirect effects of the pandemic.^26,39,40,41,42^ Telehealth may also have allowed clinicians to provide ongoing and expanded access to behavioral health services relative to other services,^43,44^ where in-person physical examination is often not required. Observed increases in 2020 STI visit volumes at FQHCs differ somewhat from other 2020 literature^45,46^; however, these increases may have been driven by heightened cases in the latter half of 2020, as observed in national reporting but which is not testable in the present data.^47^

Findings from this study have important implications for policy and practice. First, health systems must compensate for care missed during the COVID-19 pandemic, including missed preventive screenings and insufficient management of chronic conditions. Second, health systems must also meet the increased demand for behavioral health services. To accomplish these 2 goals, extension of funding will likely be necessary going forward to ensure adequate resources and staffing to address pent-up demand, meet new demand, mitigate staffing retention and burnout issues, support outreach needs, and expand quality-improvement infrastructure. While more than $9 billion in federal funding was available exclusively to FQHCs during the pandemic, this funding was primarily aimed at keeping FQHCs open and operating during this time.^17,18,19^ Extended FQHC funding can further support efforts to hire staff who are able to engage in targeted outreach to patients who missed recommended care during this period, especially to patient populations who already experience health inequities, which will be important for ensuring that inequities are not further exacerbated.

Moreover, observed reductions in visit volumes in 2020—which persisted into 2021 for some types of visits—translate into less patient revenue for FQHCs, given that most FQHCs are reimbursed on a per-visit basis.^48^ These revenue losses, while likely partially mitigated through federal relief funds, come at a time when more financial revenue is needed to invest in expanded quality-improvement infrastructure and staffing. Thus, the experience of the COVID-19 pandemic suggests that the current visit-based reimbursement model for FQHCs may leave FQHCs financially vulnerable during times of unexpected change or crisis. Expanded implementation of alternative payment models at FQHCs could help mitigate this risk.

Finally, given stark changes in quality measures during the COVID-19 pandemic, quality measurement and value-based care models will need to adapt to the shorter- and longer-term effects of the pandemic on quality measures.^49^ This may be particularly important for accountable care organizations, managed care plans, and other health system entities whose finances or operations are closely linked with quality-measure performance. While the Centers for Medicare & Medicaid Services and most states granted flexibilities for value-based payment models and quality reporting during the pandemic, including opting out of quality reporting during periods affected by COVID-19 and removing downside risk in some programs, flexibilities were largely temporary.^49,50,51^ However, the pandemic could continue to influence quality-of-care measures for years. These findings highlight examples of measures that may require targeted quality-improvement efforts in subsequent years and/or continued flexibilities in how measures are used for rewarding performance.

### Limitations

It is important to interpret this study’s findings in the context of its limitations. First, these are descriptive findings only. We do not test the effect of specific system changes or policies on quality or service volume. Second, data are reported at the FQHC level, which precludes us from examining changes in individual care-seeking behavior. Third, while the UDS is the only data source that includes the universe of US FQHCs, there is potential for measurement and reporting error, especially given competing COVID-19 reporting demands coupled with staffing constraints. Nonetheless, the direction of error is likely to be random and, therefore, is unlikely to systematically bias the present results. Future studies should validate these findings at the patient level using electronic health record data. Fourth, because quality-of-care measures required 1 medical visit during the measurement period to qualify for the denominator, the quality findings likely underestimate the magnitude of decrease in measured quality within the true FQHC patient population. Fifth, these results capture average changes across FQHCs, although there may be important heterogeneity in trends across different types of FQHCs. Finally, these findings represent changes from the first 2 years of the pandemic only. It will be important to continue monitoring outcomes over time to assess longer-term changes in measured quality and service volume.

## Conclusions

In this cohort study of US FQHCs, visit volumes for preventive care, chronic disease care, follow-up care, eye examinations, and oral examinations declined considerably during the first year of the COVID-19 pandemic, and most of these visit types did not return to prepandemic levels by 2021. In contrast, visits for mental health and substance use diagnoses increased in 2020 to 2021. Nearly all quality-of-care HEDIS measures at FQHCs declined in 2020, and nearly all remained below prepandemic levels in 2021. These findings suggest that sustained federal funding to FQHCs is needed to expand service capacity, staffing, and patient outreach to compensate for missed care during the pandemic, as well as to meet increased demand for behavioral health needs likely exacerbated by the pandemic. Quality reporting and value-based care models must also adapt to the shorter- and longer-term effects of the pandemic on quality measures.

## Notes

Supplement 1. **eTable 1.** Quality of care measure definitions **eTable 2.** Characteristics of study sample FQHCs vs. FQHCs excluded due to newly opening during study period (2021) **eTable 3.** Year-over-year changes in quality of care measures at FQHCs compared to 2019 levels **eFigure 1.** Trends in early entry to prenatal care at FQHCs (2016-2021) **eFigure 2.** Trends in normal birthweight among infants born to pregnant patients at FQHCs (2016-2021)

Supplement 2. Data Sharing Statement
