# Supplementary material for: Changes in Performance Measures and Service Volume at US Federally Qualified Health Centers During the COVID-19 Pandemic
Source: JAMA Health Forum. 2023 Apr 7;4(4):e230351. doi: 10.1001/jamahealthforum.2023.0351 (PMC10082403; doi:10.1001/jamahealthforum.2023.0351)
Supplement: Supplement 1. — eTable 1. Quality of care measure definitions eTable 2. Characteristics of study sample FQHCs vs. FQHCs excluded due to newly opening during study period (2021) eTable 3. Year-over-year changes in quality of care measures at FQHCs compared to 2019 levels eFigure 1. Trends in early entry to prenatal care at FQHCs (2016-2021) eFigure 2. Trends in normal birthweight among infants born to pregnant patients at FQHCs (2016-2021) [file jamahealthforum-e230351-s001.pdf]

Supplemental Online Content

Cole MB, Lee EK, Frogner BK, Wright B. Changes in performance measures and service volume at US federally qualified health centers during the COVID-19 pandemic. *JAMA Health Forum*. 2023;4(4);e230351. doi:10.1001/jamahealthforum.2023.0351

eTable 1. Quality of care measure definitions

eTable 2. Characteristics of study sample FQHCs vs. FQHCs excluded due to newly opening during study period (2021)

eTable 3. Year-over-year changes in quality of care measures at FQHCs compared to 2019 levels

eFigure 1. Trends in early entry to prenatal care at FQHCs (2016-2021)

eFigure 2. Trends in normal birthweight among infants born to pregnant patients at FQHCs (2016-2021)

This supplemental material has been provided by the authors to give readers additional information about their work.

© 2023 Cole MB et al. *JAMA Health Forum.*

eTable 1. Quality of Care Measure Definitions

Measure	Definition
**Process measures**	
Cervical Cancer Screening	Percentage of women 21-64 years of age who were screened for cervical cancer.
BMI Screening and Follow-Up Plan (Adults)	Percentage of patients 18 years of age and older with (1) BMI documented and (2) follow-up plan documented if BMI is outside normal parameters.
Weight Assessment and Counseling for Nutrition and Physical Activity (Children and Adolescents)	Percentage of patients 3–17 years of age who had an outpatient medical visit and who had evidence of height, weight, and body mass index (BMI) percentile documentation ***and*** who had documentation of counseling for nutrition ***and*** who had documentation of counseling for physical activity during the measurement period.
Use of Aspirin or Antiplatelet in Ischemic Vascular Disease (IVD)	Percentage of patients aged 18 years of age and older who were diagnosed with acute myocardial infarction (AMI), or who had a coronary artery bypass graft (CABG) or percutaneous coronary interventions (PCIs) in the 12 months prior to the measurement period, ***or*** who had an active diagnosis of IVD during the measurement period, and who had documentation of use of aspirin or another antiplatelet during the measurement period.
Colorectal Cancer Screening	Percentage of patients 50 through 75 years of age who had appropriate screening for colorectal cancer.
Early Entry into Prenatal Care	Percentage of prenatal care patients who entered prenatal care during their first trimester.<br>**First Trimester**: Report patients who were prenatal patients during the reporting period and whose first visit occurred when they were estimated to be pregnant up through the end of the 13th week after the first day of their last menstrual period.<br>**Second Trimester**: Report patients who were prenatal patients during the reporting period whose first visit occurred when they were estimated to be between the start of the 14th week and the end of the 27th week after the first day of their last menstrual period.<br>**Third Trimester**: Report patients who were prenatal care patients during the reporting period and whose first visit occurred when they were estimated to be 28 weeks or more after the first day of their last menstrual period.
Tobacco Use Screening and Cessation Intervention	Percentage of patients aged 18 years of age and older who (1) were screened for tobacco use one or more times within 24 months, ***and*** (2) if identified to be a tobacco user received cessation counseling intervention.
Depression Screening and Follow-Up Plan	Percentage of patients aged 12 years and older screened for depression on the date of the visit or 14 days prior to the visit using an age-appropriate standardized

	depression screening tool ***and***, if positive, had a follow-up plan documented on the date of the visit.
Dental Sealants for Children	Percentage of children, age 6–9 years, at moderate to high risk for caries who received a sealant on a first permanent molar during the measurement period.
**Intermediate outcome measures**	
Blood Pressure Control in Hypertensive Patients	Percentage of patients 18-85 years of age who had a diagnosis of hypertension overlapping the measurement period and whose most recent blood pressure (BP) was adequately controlled (less than 140/90 mmHg) during the measurement period.
HbA1c Control in Diabetic Patients	Percentage of patients 18-75 years of age with diabetes who had hemoglobin A1c (HbA1c) greater than 9.0 percent during the measurement period.
Normal birthweight for Infants Born to Pregnant Patients	Weight at birth was equal to or greater than 2,500 grams.
Note: Definitions as of 2020 – please see definitions by HRSA available at: https://bphc.hrsa.gov/sites/default/files/bphc/funding/2020-uds-manual.pdf	

© 2023 Cole MB et al. *JAMA Health Forum.*

eTable 2. Characteristics of study sample FQHCs vs. FQHCs excluded due to newly opening during study period (2021)

FQHC Characteristic	**FQHCs excluded due to newly opening in study period** (N=218 FQHCs, representing 2.5 million patients)	**Study sample FQHCs** (N=1037 FQHCs, representing 26.6 million patients)
Age group, %		
Children (<18)	20.7%	24.1%
Adults (18-64)	65.4%	61.9%
Elderly (>=65)	13.9%	14.0%
Female, %	55.7%	56.4%
Race/ethnicity, %		
Black, non-Hispanic	17.7%	18.9%
Hispanic	29.0%	28.1%
White, non-Hispanic	40.7%	41.1%
Other race,[a] non-Hispanic	12.6%	11.8%
Primary language other than English, %	20.5%	19.1%
Insurance coverage type, %		
Medicaid	44.3%	42.9%
Uninsured	23.7%	22.1%
Private	20.7%	22.2%
Medicare	10.5%	12.3%
Other	0.7%	0.6%
Poverty level, %		
Under 100% FPL	60.9%	63.2%
100-200% FPL	26.1%	25.0%
>200% FPL	13.0%	11.8%
Rural, %	31.9%	44.3%
Without housing, %	7.2%	7.4%
Unique patients served/FQHC (mean, SD)	11560 (13390)	25667 (29743)

Abbreviations: FQHC federally qualified health center; FPL federal poverty level; SD standard deviation.
[a] Other race includes patients who identify as American Indian or Alaskan Native, Asian, Native Hawaiian or Other Pacific Islander, or more than one race.

© 2023 Cole MB et al. *JAMA Health Forum.*

eTable 3. Year-over-year changes in quality of care measures at FQHCs compared to 2019 levels

Abbreviations: FQHC, federally qualified health center; BMI, body mass index; IVD, Ischemic Vascular Disease; HbA1c, hemoglobin A1C

	2016 vs. 2019[a]		2017 vs. 2019[a]		2018 vs. 2019[a]		2020 vs. 2019[a]		2021 vs. 2019[a]	
	Coefficient for absolute percentage point difference (95% CI)	*P* value	Coefficient for absolute percentage point difference (95% CI)	*P* value	Coefficient for absolute percentage point difference (95% CI)	*P* value	Coefficient for absolute percentage point difference (95% CI)	*P* value	Coefficient for absolute percentage point difference (95% CI)	*P* value
**Process measures**										
Cervical Cancer Screening	-1.83 (-2.66, -0.99)	<0.001	-0.61 (-1.32, 0.99)	0.092	-0.49 (-1.04, 0.06)	0.078	-3.76 (-4.32, -3.20)	<0.001	-2.42 (-3.06, -1.77)	<0.001
BMI Screening and Follow-Up Plan (Adults)	-9.85 (-1.11, -8.60)	<0.001	-9.19 (-10.3, -8.60)	<0.001	-2.87 (-3.59, -2.15)	<0.001	-5.63 (-6.54, -4.72)	<0.001	-8.87 (-10.0, -7.72)	<0.001
Weight Assessment and Counseling (Children)	-8.59 (-10.1, -7.06)	<0.001	-5.67 (-7.02, -4.32)	<0.001	-6.38 (-7.51, -5.25)	<0.001	-6.38 (-7.51, -5.25)	<0.001	-2.34 (-3.60, -1.07)	<0.001
Use of Aspirin or Antiplatelet in Ischemic Vascular Disease (IVD)	-2.41 (-3.37, -1.45)	<0.001	-2.12 (-2.96, -1.28)	<0.001	0.19 (-0.52, 0.89)	0.602	-2.04 (-2.66, -1.42)	<0.001	-2.42 (-3.12, -1.72)	<0.001
Colorectal Cancer Screening	-5.16 (-6.06, -4.27)	<0.001	-3.28 (-3.98, -2.57)	<0.001	-1.72 (-2.25, -1.19)	<0.001	-3.30 (-3.99, -2.62)	<0.001	-2.31 (-3.10, -1.52)	<0.001
Early Entry into Prenatal Care	-0.43 (-1.45, -0.60)	0.413	1.48 (-0.70, 0.99)	0.732	-0.65 (-1.42, 0.11)	0.093	0.25 (-0.66, 1.16)	0.593	-0.008 (-1.07, 1.05)	0.988
Tobacco Use Screening and Cessation Intervention	-1.74 (-2.72, -0.76)	0.001	0.96 (0.15, 1.78)	0.021	1.41 (0.72, 2.10)	<0.001	-3.71 (-4.46, -2.96)	<0.001	-4.38 (-5.30, -3.45)	<0.001
Depression Screening and Follow-Up Plan	-10.6 (-11.9, -9.27)	<0.001	-5.49 (-6.64, -4.35)	<0.001	-2.05 (-2.87, -1.23)	<0.001	-6.97 (-7.99, -5.94)	<0.001	-3.98 (-5.10, -2.86)	<0.001
Dental Sealants for Children	-9.97 (-11.9, -7.98)	<0.001	-7.45 (-9.41, -5.54)	<0.001	-3.81 (-5.36, -2.27)	<0.001	-5.03 (-6.68, -3.38)	<0.001	-0.25 (-2.09, 1.58)	0.789
**Intermediate outcome measures**										
Blood Pressure Control in Hypertensive Patients[b]	-1.82 (-2.39, -1.26)	<0.001	-1.62 (-2.11, -1.14)	<0.001	-1.25 (-1.64, -0.85)	<0.001	-6.50 (-6.99, -6.01)	<0.001	-4.39 (-4.91, -3.86)	<0.001
HbA1c Control in Diabetic Patients[b]	-0.54 (-1.23, 0.16)	0.132	-1.49 (-2.09, -0.89)	<0.001	-1.03 (-1.55, -0.52)	<0.001	-3.89 (-4.44, -3.34)	<0.001	-0.83 (-1.35, -0.31)	0.002
Normal birthweight for Infants Born to Pregnant Patients	0.21 (-0.95, 1.37)	0.724	-0.36 (-1.48, 0.76)	0.533	0.35 (-0.66, 1.36)	0.499	1.19 (0.40, 1.99)	0.003	0.65 (-0.25, 1.55)	0.159

[a] Results compare absolute changes in the percentage of eligible patients achieving the quality measure in each individual year compared to 2019 (the reference year)

[c] Blood pressure (BP) and HbA1C control measures require (1) clinical control (i.e. most recent BP <140/90 mmHg and HbA1c <9%) and (2) having a documented measurement in the study period. Patients who qualify for the denominator but who do not have a documented measurement are classified as not having control.

© 2023 Cole MB et al. *JAMA Health Forum.*

eFigure 1. Trends in early entry to prenatal care at FQHCs (2016-2021)

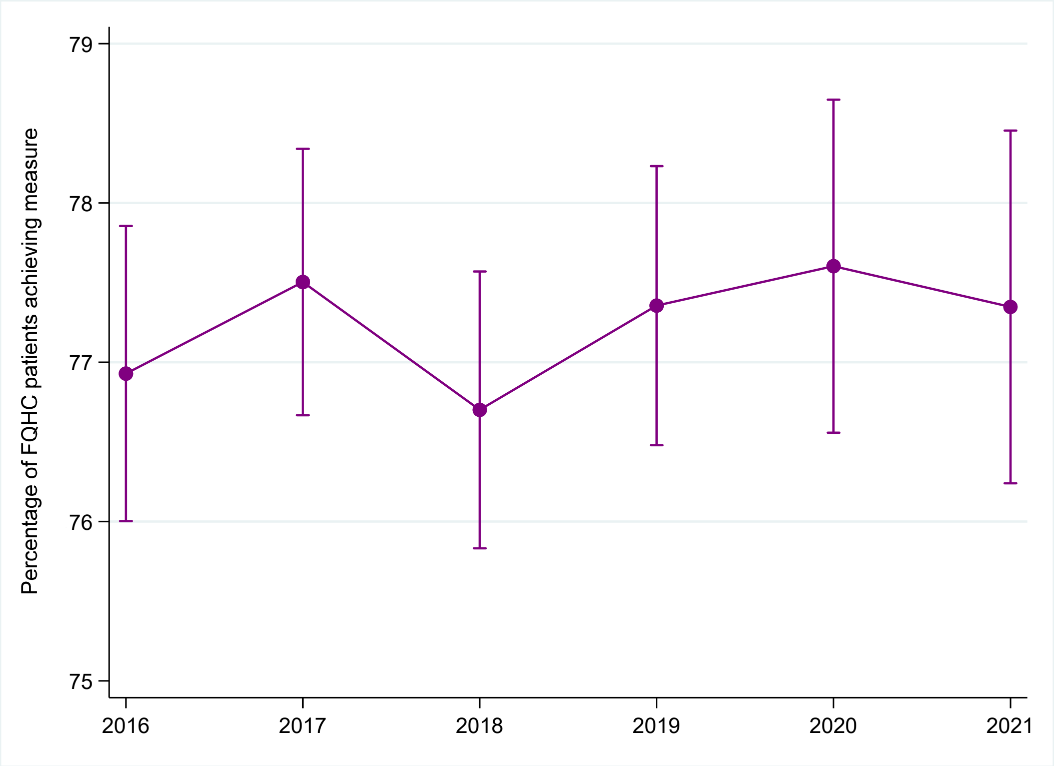

© 2023 Cole MB et al. *JAMA Health Forum.*

eFigure 2. Trends in normal birthweight among infants born to pregnant patients at FQHCs (2016-2021)

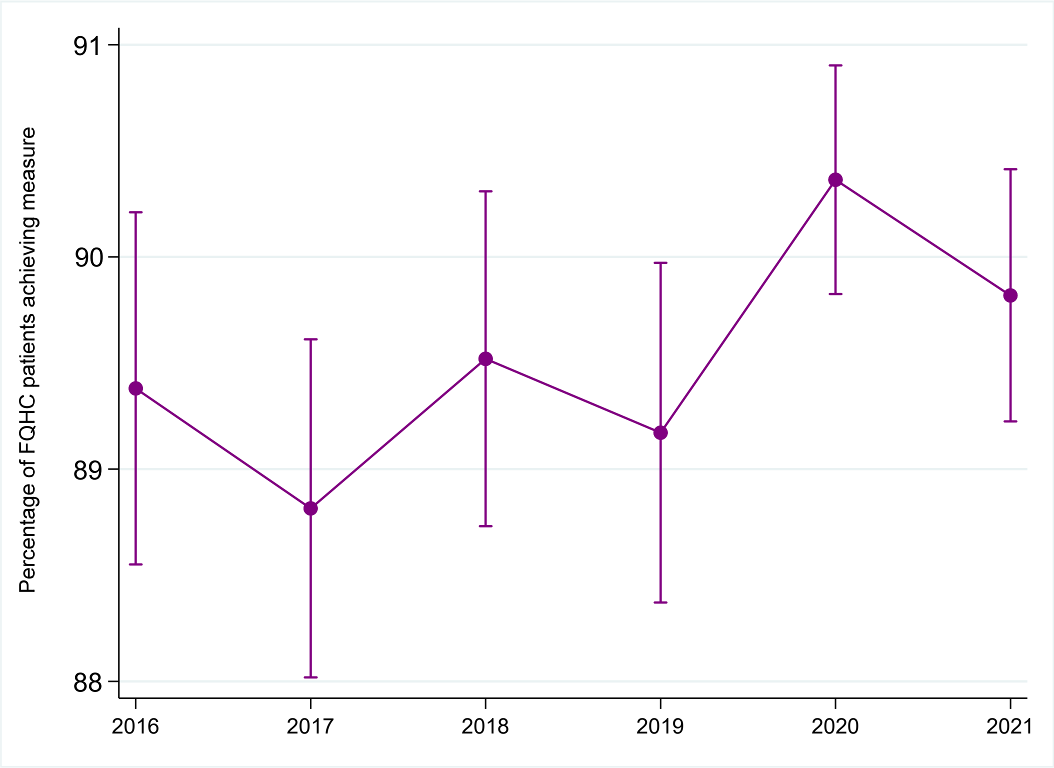

© 2023 Cole MB et al. *JAMA Health Forum.*
