# Supplementary material for: Changes in Performance Measures and Service Volume at US Federally Qualified Health Centers During the COVID-19 Pandemic
Source: JAMA Health Forum. 2023 Apr 7;4(4):e230351. doi: 10.1001/jamahealthforum.2023.0351 (PMC10082403; doi:10.1001/jamahealthforum.2023.0351)
Supplement: Supplement 2. — Data Sharing Statement [file jamahealthforum-e230351-s002.pdf]

Data Sharing Statement

Cole. Changes in Performance Measures and Service Volume at US Federally Qualified Health Centers During the COVID-19 Pandemic. *JAMA Health Forum*. Published April 07, 2023. doi:10.1001/jamahealthforum.2023.0351

Data
Data available: No

Additional Information
Explanation for why data not available: Some data elements are protected by a DUA. Limited data files can be made available upon request.
